# A New Approach to Detection of Systematic Errors in Secondary Substation Monitoring Equipment Based on Short Term Load Forecasting

**DOI:** 10.3390/s16010085

**Published:** 2016-01-12

**Authors:** Javier Moriano, Francisco Javier Rodríguez, Pedro Martín, Jose Antonio Jiménez, Branislav Vuksanovic

**Affiliations:** 1 Department of Electronics, University of Alcalá, Alcalá de Henares, Madrid 28805, Spain; javier.moriano@depeca.uah.es (J.M.); martin@depeca.uah.es (P.M.); jimenez@depeca.uah.es (J.A.J.); 2 School of Engineering, University of Portsmouth, Winston Churchill Ave, Portsmouth PO1 3HJ, UK; branislav.vuksanovic@port.ac.uk

**Keywords:** Short Term Load Forecasting (STLF), Artificial Neural Network (ANN), measurement error detection, secondary substation (SS)

## Abstract

In recent years, Secondary Substations (SSs) are being provided with equipment that allows their full management. This is particularly useful not only for monitoring and planning purposes but also for detecting erroneous measurements, which could negatively affect the performance of the SS. On the other hand, load forecasting is extremely important since they help electricity companies to make crucial decisions regarding purchasing and generating electric power, load switching, and infrastructure development. In this regard, Short Term Load Forecasting (STLF) allows the electric power load to be predicted over an interval ranging from one hour to one week. However, important issues concerning error detection by employing STLF has not been specifically addressed until now. This paper proposes a novel STLF-based approach to the detection of gain and offset errors introduced by the measurement equipment. The implemented system has been tested against real power load data provided by electricity suppliers. Different gain and offset error levels are successfully detected.

## 1. Introduction

In the past few decades, significant advances in communication and information technology have accelerated the development and introduction of new broadband communication technologies in power systems. This fact has facilitated the power system automation in primary and secondary substations. With the aim of taking advantage of the current technology, there exists the IEC 61850 global standard for substation automation which is becoming particularly popular in recent years [1].

Nowadays, the development of Electronic Instrumental Transducer (EIT) and the importance of communication and information technology are quickly gaining acceptance in SSs. Consequently, traditional Substation Automation System (SAS) where Intelligent Electronic Devices (IEDs) were hardwire-linked are being challenged by this new protocol standardized as IEC 61850. Different EITs carry out the necessary current and voltage measurements, which are used to control and protect the SSs. However, the advantages that this approach brings could be negatively affected by the existence of erroneous measurements in the signal acquisition process [2].

On the other hand, the load profile and, therefore, the current consumption are strongly related to their historical measurement data depending on different variables, such as economic factors and environmental data. With the purpose of continuously maintaining the electricity generation and consumption balance, several approaches to load forecasting have been proposed during the last few years and some good examples can be found in [3,4].

In this paper, by using load forecasting techniques and comparing the forecast load with that from the measurement equipment in the SSs, different systematic error levels can be estimated, namely gain and offset errors. The implemented system is tested against data from both an SS simulator and electricity suppliers.

The scope of this work falls within two research fields: (1) short term load forecasting and (2) erroneous and false measurement detection. With this in mind, what follows presents the literature on both fields.

Different forecasting and prediction models have been employed in several fields, such as wind power prediction models [5] and photovoltaic power forecasting models [6]. As far as load forecasting is concerned, in literature, various approaches have been proposed to solve this issue. Load forecasting involves estimating the future electric load, for a forecast horizon, based on the available information about the state of the system. In this regard, this paper focuses on short term load forecast (STLF) to estimate the load with a horizon from one hour to one week. In [7], authors propose a new neural network approach to STLF based on a new modified learning algorithm. The results obtained are compared with those from other works [8,9] with the aim of evaluating the validity of the proposed approach. In [10,11], environmental variables are also employed, whereas in [12,13] only the historical load consumption is considered without a detrimental impact on the accuracy. The latter is the strategy adopted in this paper. In [12], the authors propose a solution for STLF in microgrids based on a three-stage architecture, which consists of a Self Organizing Map (SOM), a clustering via k-means algorithm, and a Multilayer Perceptron (MLP). A set of 29 inputs are provided to the MLP: 24 inputs that represent the hourly load of the previous day; two inputs for the day of the week of the previous day and two inputs for the month of the previous day in the form of sines and cosines; and lastly, one more input that represents the next day total load estimation. Finally, a total of 24 outputs corresponding to the estimated load for the forecasting day (d) are obtained.

In [13], a different approach to one-day ahead load profile prediction is presented. The mean absolute percentage of the forecasting error is reduced between 0.5% and 16% by employing an Artificial Neural Network (ANN). Three different scenarios with different inputs are considered. The inputs to the ANN depend on the model implemented. For example, Model I requires 72 inputs, namely: hourly load values of the last day available, load values of the same weekday of previous week and load values of the same weekday two weeks before. The authors define a second model (Model II) by including three more inputs: two inputs represent the day of the week and the third one is used to distinguish between working day or vacation. In both models, 24 outputs are obtained which form the hourly predicted load for the forecast day. These outputs along with the 72 inputs of the first model could also be used as input of a new ANN with a total of 96 inputs (Model III).

Regarding erroneous measurement detection, this problem has been addressed in different papers. For example, in [14], the sensor failure detection is discussed employing a neural network. In [15] a method based on fractal dimension is implemented for sensor fault diagnosis. In [16], nonlinear principal component analysis (NLPCA) method for faulty measurement reconstruction is proposed. Finally, in [17], another neural-network-based algorithm is employed for sensor failure detection for a flight control system.

Focusing on SSs, in [2], pattern identification is utilized to detect erroneous measurements. In an attempt to achieve high pattern identification precision within the time limit imposed by the protection systems, a Radial Basis Function Neural Network (RBFNN) and an Orthogonal Least Square (OLS) learning algorithm are implemented. The neural network calculates the probability of fault occurrence based on measurements from all the EITs.

In order to provide a way of detecting a malfunction of an EIT in a particular SS, this work proposes a new approach to measurement error detection based on STLF. As mentioned before, in the literature, there are many examples of using STLF for monitoring and planning purposes and various approaches have been proposed to solve this issue. Interestingly, STLF has not been used for error detection until now. This strategy represents an innovative approach to error detection in SSs. This strategy greatly facilitates the detection of gain and offset error, thereby allowing preventive maintenance in the SSs to be performed. Particularly, the system presented in this paper could be effectively employed in open SS Nodes that are described in [18,19]. The open SS node is based on an architecture that combines smart metering with grid automation. While the EIT is operating, the developed system allows errors to be detected. In the event of occurring an error level higher than a threshold, an alarm can be raised and sent to the control center. Further tests for verifying the scope of the error could then be conducted by an operator.

This paper is divided into four sections. After the introduction, in Section 2, a global description of the system and the adopted methods are described. Section 3 analyzes both the data used to test the implemented system and the results achieved. Finally, some conclusions are drawn and future works are described in the final section.

## 2. Methodology and System Description

**Figure 1:**
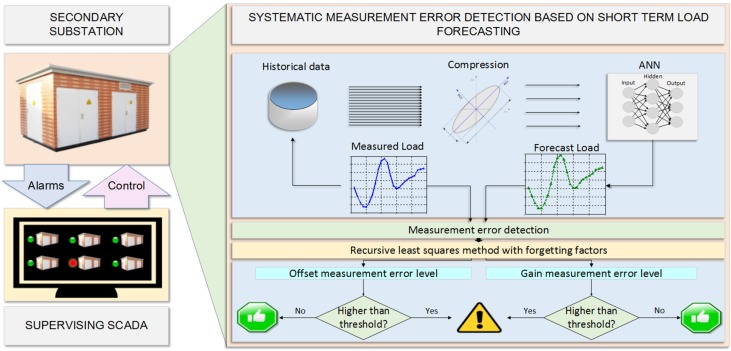
Overview of the general system.

In this section, a general view on the employed system is discussed. Figure 1 shows an overview of the system. The available information pertains to the historical measurements from different SSs. The different electrical variables measured are: active and reactive power, voltage and current. However, for the system under study, just the current historical measurements are considered. This is due to the fact that the equipment for current measurement is more prone to errors on account of the considerable variability of the current.

The algorithm is divided into two steps: load forecasting and error estimation. Firstly, the available data is used to provide one-day ahead load forecast on a hourly basis. Secondly, once the measured load for the day under consideration is available, the error level is estimated by comparing both data sets, *i.e.*, forecast and measured load.

### 2.1. Load Forecasting

As mentioned previously, the load forecasting model uses only historical measurement data, which consists of measurements from a set of SSs. Following one of the methods employed in [13], the system effectively performs, providing an accurate forecast. The chosen data, whereby the load profile on an hourly basis is estimated, is listed below:

- 24 h load of the previous available day (d-1)
- 24 h load of the previous week same day (w-1) (d)
- 24 h load of the two previous weeks same day (w-2) (d)
- Day of the week (sine and cosine)
- Month of the year (sine and cosine)

Therefore, a total of 72 samples corresponding to hourly load are used. Since there exists a strong correlation among the 72 samples, a principal component analysis (PCA) procedure can be used to reduce the number of dimensions. This technique has been successfully employed in different fields such as face recognition [20], voice processing [21] and in ultrasonic sensors [22]. PCA basically is a statistical approach that linearly transforms an original set of variables into a smaller set of uncorrelated variables, which represent the largest variance from the multivariate input data.

The PCA algorithm has been described in [22,23]. Given a matrix *X* with dimension M×N, the first step consists in subtracting the mean of the elements in a particular row from each element of that row. Then, the covariance matrix from the previous matrix with the subtracted mean is obtained (N×N) and the eigenvectors and eigenvalues for that matrix are calculated. The eigenvectors with the highest eigenvalues represent the principal components (*k*), and the number of principal components need to be determined. The set of eigenvectors corresponding to the principal components form the feature matrix *V* and that can be expressed as:

(1) $$V={\left[\lambda_{1},\lambda_{2},\ldots ,\lambda_{k}\right]}_{\left(N\times k\right)}$$

Finally, the new compressed matrix (Y) is derived by multiplying the transposed feature matrix with the transposed zero-mean matrix previously calculated, as shown in the following equation:

(2) $$Y={\left[V^{T}\cdot {\bar{X}}^{T}\right]}_{\left(k\times M\right)}$$

**Figure 2:**
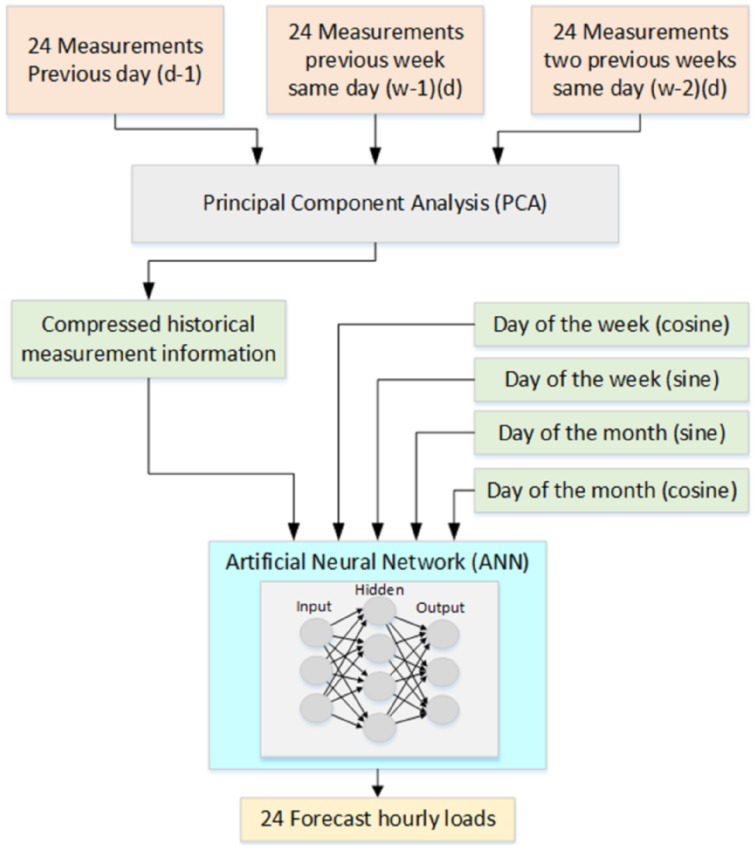
Functional diagram of the load forecasting system.

The principal components resulting from the PCA process are the inputs of the ANN along with the data that represents the day of the week and the month of the year. Figure 2 shows the functional diagram of the system.

As mentioned before, a set of 72-dimensional data provides historical information for the forecasting process of forecasting. Bearing in mind that the training process can be lengthy, the smaller the data set, the faster the process will be. Due to the strong correlation among the inputs, this data set can be described with only a few most significant components that hold 97% of the information, *i.e.*, 97% of the variance for the principal components selected. In the system discussed in this paper, the number of principal components depends on whether the input data come from the simulator or from real measurements. This value ranges from seven to 20 components, being higher for the real data on account of less correlation.

The ANN used in this work is a one-hidden layer perceptron since it is the most frequently employed in forecasting and time series due to its performance [24]. The output and the input signals are related by the following equation:

(3) $$y=\varphi \left(\sum_{j=1}^{n}x_{j}\cdot w_{j}-\theta \right)$$

where *y* is the output, $x_{j}$ represents the input data, $w_{j}$ denotes the weight that is associated with each $x_{j}$, and *θ* is the threshold. Finally, φ is the transfer function that is usually expressed as:

(4) $$\varphi =\frac{1}{1+e^{-x}}$$

**Figure 3:**
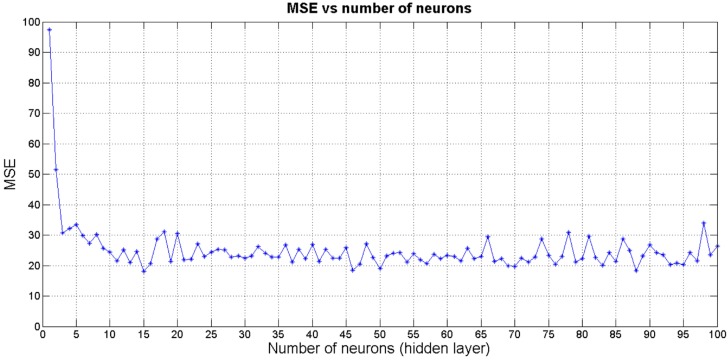
MSE depending on the number of neurons.

An important issue is to define the number of neurons of the hidden layer since this significantly affects the performance of the network in terms of execution time and accuracy. In this regard, an iterative process, which consists in carrying out different tests is used. Varying the number of neurons of the hidden layer from 1 to 100 and comparing the performance for each iteration, the ANN that minimizes the test error is composed of a 15-neuron hidden layer. Figure 3 shows the obtained mean square error (MSE) depending on the number of neurons in the hidden layer.

The Levenberg–Maquardt algorithm has been chosen for the ANN training for two reasons: (i) the number of iterations required for the ANN training is smaller in comparison to other techniques; (ii) this algorithm always guarantees the convergence of the training process [25,26].

Taking into consideration that every EIT to be checked by the described system generates its own data, the ANN differs between the different EITs, hence the importance of selecting the algorithm that best provides accuracy and fast convergence in equal measure.

### 2.2. Error Measurement

Once the load forecasting has been carried out and the new measurements for the day (d) are available, the process of error measurement detection for the different EITs can start. As stated in the introduction, the errors to be detected are systematic [27] and can be classified into two different types, namely: offset and gain errors.

**Figure 4:**
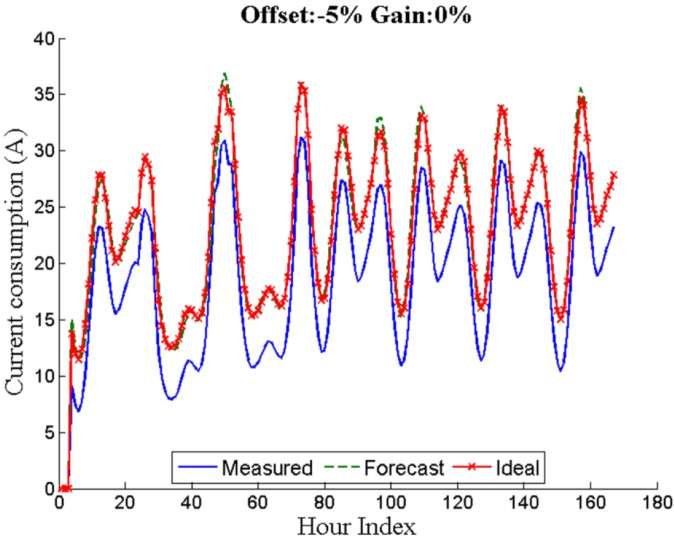
Seven days with an offset error of −5%.

Offset errors affect each acquired sample in a systematic way and hence they can question the accuracy of the measurements in the SS. The offset error in percentage is compared against the full scale. Figure 4 shows the load measurement deviation due to the offset.

**Figure 5:**
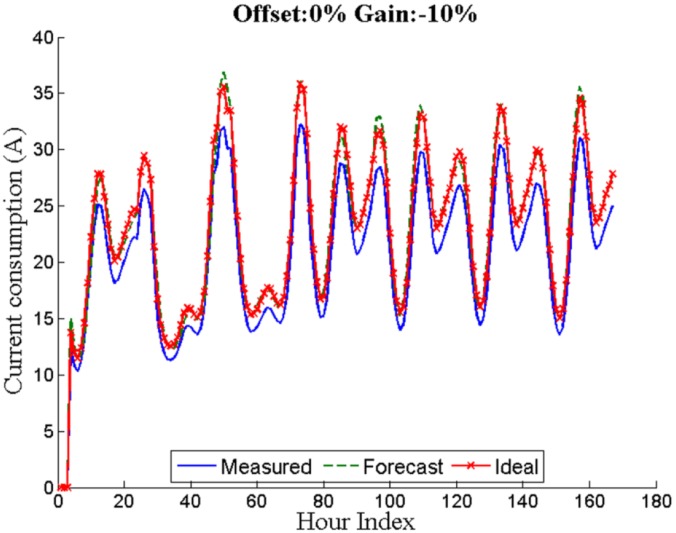
Seven days with a gain error of −10%.

Unlike offset errors, gain errors are multiplicative and affect all the measurements proportionally. Figure 5 shows a comparison between the measured, the forecast and the ideal load over a period of seven days for a gain error percentage of −10%.

Regarding their presence, offset and gain errors can occur simultaneously, which greatly hinders the error detection. In this context, both errors must be detected independently. Interestingly, the system described in this paper performs well under worst-case scenarios such as the occurrence of both errors with different signs. In this context, the most general possible scenario is considered when both errors appear at the same time as described in the following equation:

(5) $$m_{lp}\left(t\right)=\left(i_{lp}\left(t\right)\cdot \alpha \right)+\beta$$

where $m_{lp}$ represents the measured load profile, $i_{lp}$ is the ideal measurement load profile, *i.e.*, when no error occurs, and *α* and *β* represent the gain and offset error, respectively.

By using the strategy described previously, the forecast load profile ($f_{lp}\left(t\right)$) can be estimated. Since the process of forecasting introduces a random error ($e_{f}\left(t\right)$), Equation (5) can be rewritten as:

(6) $$\begin{array}{ccc} m_{lp}\left(t\right) & = & (\left(f_{lp}\left(t\right)+e_{f}\left(t\right)\right)\cdot \alpha )+\beta \end{array}$$

(7) $$\begin{array}{ccc} & = & f_{lp}\left(t\right)\cdot \alpha +e_{f}\left(t\right)\cdot \alpha +\beta \end{array}$$

It goes without saying that the better the load prediction, the smaller the error introduced. However, this error can be negligible on account of the fact that it is a random error with mean 0. Therefore, by using different averaging processes, Equation (7) can be reformulated as:

(8) $$\bar{m_{lp}}\left(t\right)=\bar{f_{lp}}\left(t\right)\cdot \alpha +\beta$$

Differentiating both terms in Equation (8) leads to Equation (9):

(9) $$\frac{\Delta \bar{m_{lp}}\left(t\right)}{\Delta t}=\frac{\Delta (\bar{f_{lp}}\left(t\right)\cdot \alpha +\beta )}{\Delta t}=\frac{\Delta \bar{f_{lp}}\left(t\right)}{\Delta t}\cdot \alpha$$

Then, the following equation can be obtained by solving for *α*:

(10) $$\alpha =\frac{\Delta {\bar{m}}_{lp}\left(t\right)/\Delta t}{\Delta {\bar{f}}_{lp}\left(t\right)/\Delta t}=\frac{\Delta {\bar{m}}_{lp}\left(t\right)}{\Delta {\bar{f}}_{lp}\left(t\right)}$$

Finally, substituting the gain error into Equation (8), and solving for *β* leads to Equation (11):

(11) $$\beta =\bar{m_{lp}}\left(t\right)-\bar{f_{lp}}\left(t\right)\cdot \alpha$$

The proposed system is able to estimate the value of gain and offset error independently. Since there exists a considerable variability in the offset and gain measurement error estimation, the recursive least squares algorithm with forgetting factors (FRLS) is employed for filtering. This algorithm has been extensively utilized in the time-varying system as in [28] where a novel FRLS is developed. To be more precise, in this paper, a forgetting factor of 0.83 has been employed due to the performance enhancement on the trade-off between filtering and fast response.

## 3. Simulations and Experimental Results

With the aim of testing the system presented in this paper, two different data sets are used, namely: simulated data set from a simulator, and real data from SSs.

As far as the first data set is concerned, the simulator has been developed by a distribution operator, and it provides simulated measurements from different SSs located in the Henares Corridor, in Madrid. This simulator provides four measurements: active and reactive power, voltage and current. As mentioned in the introduction, only the current measurement is considered given that the equipment for current measurement is more prone to errors. Nevertheless, this system can be applied to the rest of the electrical variables.

A simulated data set based on current measurement is thus obtained every 15 min for two years (from 1 January 2010 to 31 December 2011).

On the other hand, the system has also been tested against a real data set obtained from different SSs located in the Community of Madrid. This data set contains information half-hourly recorded from several SSs in the period ranging from 1 January 2010 to 31 December 2011.

Although both data sets contain data acquired with different frequencies, they are analyzed in the same way since only hourly average values are taken into consideration.

Then, the data sets are split into three groups: (i) the training subset (70%), which is used to train the ANN; (ii) the validation subset (15%) used to ensure that the network is generalizing and to stop training before overfitting; and (iii) the test subset (15%) employed for a completely independent test of network generalization. Different error values of offset and gain are injected in the data set and then they are correctly detected. Likewise, the training time depends on whether the data is simulated or real. This stems from the fact that simulated data are more correlated and, therefore, fewer principal components are required.

The test was carried out in a computer with Intel Core i7 CPU 860 @ 2.93GHz x8 processor and the MATLAB neural network toolbox was employed.

### 3.1. Results Based on the Simulated Data Set

**Figure 6:**
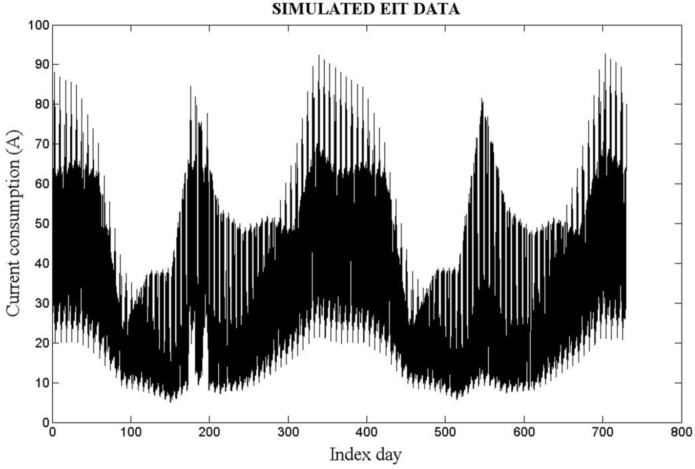
Simulated EIT—17,520 measurements obtained during 730 days.

**Figure 7:**
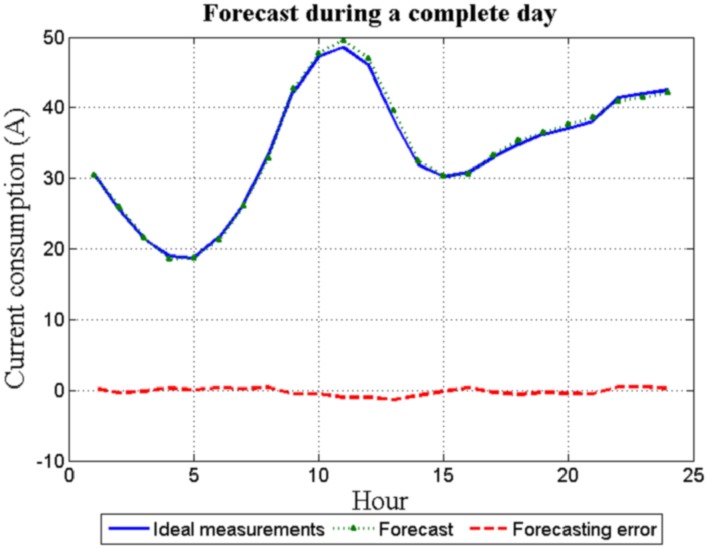
Simulated EIT—forecasting error for one-day ahead.

**Figure 8:**
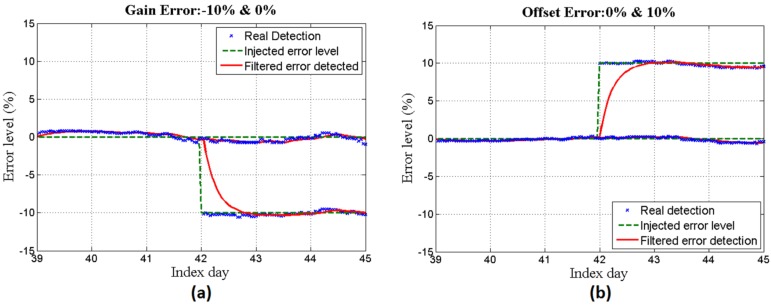
(**a**) 0% and −10% gain errors and (**b**) 0% and 10% offset errors during the days after training.

In Figure 6, Figure 7 and Figure 8, the results obtained from testing the described system against simulated data are depicted. In Figure 6, all the measurements recorded over a two-year period (730 days–17,520 measurements) are shown.

Figure 7 shows both the 24 hourly forecast load for the day *d* and the real measurements for the same day. Clearly, the error between them is negligible.

In Figure 8a,b offset and gain measurement errors are injected respectively, and the system is applied to identify the level of error. It can be seen that different values of error for gain (0% and −10%) and offset (0% and 10%) are successfully detected.

### 3.2. Results Based on the Real Data Set

**Figure 9:**
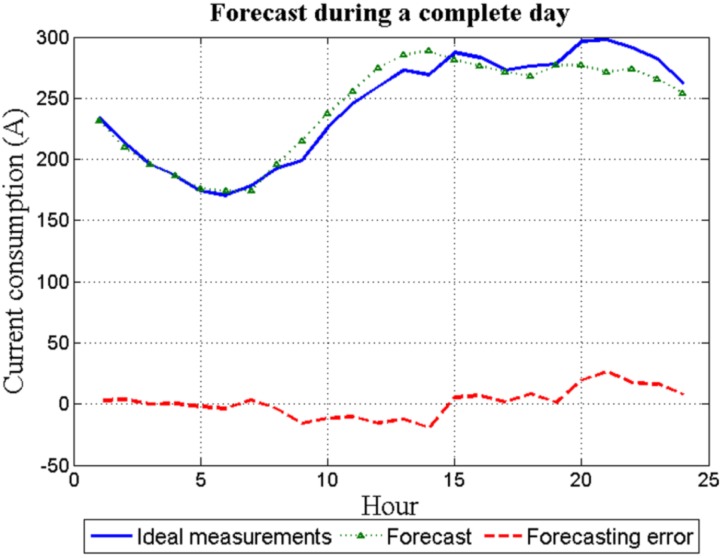
Real EIT—forecasting error for one-day ahead.

**Figure 10:**
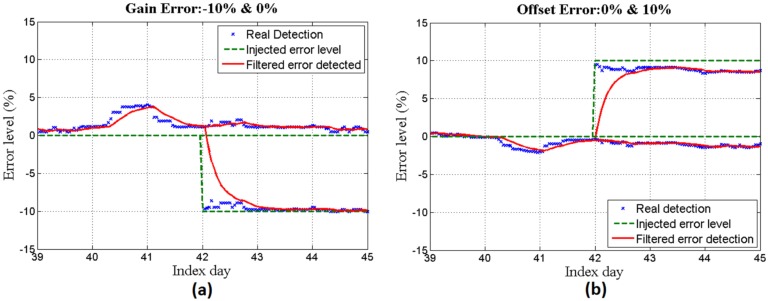
(**a**) 0 % and −10% gain errors and (**b**) 0% and 10% offset errors during the days after training.

In Figure 9 and Figure 10a,b, the results obtained from testing the described system against real data are depicted.

Figure 9 shows for the same day *d*, both the 24 hourly forecast load and the available measurements. In this case, the error between them is not negligible. However, given that the forecasting error has a random nature and due to the different averaging processes, this error does not greatly affect the system discussed in this paper.

Finally, by injecting the same error levels as was done with the simulated data set, the system is applied to identify the error levels. Figure 10a,b represent the results achieved for offset and gain error measurements, respectively.

An error analysis strategy has been implemented [29]. The mean absolute percentage error (MAPE), the normalized mean bias error (NMBE) and normalized root mean square error (NRMSE) indicators are used to compare the results. These indicators show accuracy in terms of percentage and can be expressed as:

(12) $$MAPE=\frac{1}{n}\cdot \sum_{i=1}^{n}\left|\frac{E_{i}-A_{i}}{E_{i}}\right|\times 100\;\left(\%\right)$$

(13) $$NMBE=\frac{1}{n}\cdot \sum_{i=1}^{n}\left(\frac{E_{i}-A_{i}}{E_{i}}\right)\times 100\;\left(\%\right)$$

(14) $$NRMSE=\sqrt{\frac{1}{n}\cdot \sum_{i=1}^{n}{\left(\frac{E_{i}-A_{i}}{E_{i}}\right)}^{2}}\times 100\;\left(\%\right)$$

where *n* denotes the number of samples, *E* stands for the error level injected and *A* represents the hourly detected error level.

Under normal operating conditions, the current measurement accuracy including all the equipment involved in the measuring process (mainly current transformer and measurement equipment) is below ±2%. The contribution to the error of the transformer is not higher than ±1% [30,31] and the measurement equipment [32] can introduce between ±0.5% and ±1% of error depending on the manufacturer.

Within this context, for ensuring that a systematic measurement offset or gain error is occurring without generating false alarms, a minimum threshold of ±5% is considered. Nonetheless, the results have been analyzed until a maximum measurement error of ±10%.

MAPE and NRMSE indicators show that there exists considerable variability in the result. However, the NMBE indicator is smaller, showing that the estimated error level oscillates around the right value. As a consequence, by using a recursive least squares algorithm with forgetting factor, the measured error estimation is filtered. Likewise, a comparison between the current and the filtered estimated errors in absolute value is drawn:

(15) $$abs=\left|E-\bar{F}\right|$$

where abs represents the absolute estimation error, *E* stands for the injected error level and $\bar{F}$ is the mean value of the filtered estimated error level along the different tests. Table 1 and Table 2 summarize the MAPE, NMBE, NRMSE and absolute errors indicators for gain and offset error in simulated data, respectively. Table 3 and Table 4 show the same indicators for real data.

**Table 1:** Error indicators for different gain error levels—Simulated data.

Error Level	Simulated Data—Gain			
	MAPE (%)	NMBE (%)	NRMSE (%)	Abs (%)
**−10 %**	20.915	17.021	25.857	1.705
**−9 %**	21.629	16.357	27.001	1.472
**−8 %**	22.871	15.718	28.786	1.264
**−7 %**	24.970	15.353	31.574	1.076
**−6 %**	27.958	14.930	35.393	0.895
**−5 %**	32.548	14.694	41.157	0.741
**+5 %**	34.706	11.770	42.908	0.586
**+6 %**	29.723	11.694	36.719	0.697
**+7 %**	26.193	11.641	32.346	0.814
**+8 %**	23.615	11.558	29.150	0.920
**+9 %**	21.613	11.508	26.644	1.039
**+10 %**	20.068	11.513	24.702	1.152

**Table 2:** Error indicators for different offset error levels—Simulated data.

Error Level	Simulated Data—Offset			
	MAPE (%)	NMBE (%)	NRMSE (%)	Abs (%)
**−10 %**	7.327	0.595	9.155	0.073
**−9 %**	8.146	0.903	10.170	0.085
**−8 %**	9.170	1.243	11.442	0.103
**−7 %**	10.486	1.662	13.078	0.122
**−6 %**	12.241	2.208	15.256	0.129
**−5 %**	14.664	2.839	18.269	0.140
**+5 %**	14.870	3.845	18.732	0.195
**+6 %**	12.487	3.354	15.742	0.202
**+7 %**	10.761	2.955	13.573	0.207
**+8 %**	9.455	2.633	11.931	0.211
**+9 %**	8.426	2.362	10.636	0.212
**+10 %**	7.597	2.132	9.592	0.213

**Table 3:** Error indicators for different gain error levels—Real data.

Error Level	Real Data—Gain			
	MAPE (%)	NMBE (%)	NRMSE (%)	Abs (%)
**−10 %**	29.476	11.584	35.924	1.156
**−9 %**	32.658	11.662	39.666	1.047
**−8 %**	36.603	11.560	44.344	0.925
**−7 %**	42.024	11.582	50.772	0.809
**−6 %**	48.871	11.561	58.913	0.690
**−5 %**	58.561	11.506	70.410	0.574
**+5 %**	62.436	12.890	74.999	0.641
**+6 %**	52.737	13.088	63.483	0.784
**+7 %**	45.686	13.172	55.110	0.924
**+8 %**	40.366	13.091	48.779	1.045
**+9 %**	36.504	13.218	44.210	1.188
**+10 %**	33.438	13.407	40.601	1.344

In order to carry out this test and in an attempt to enhance the versatility of this system, simulated and real data from five EITs located in different SSs is used. In this regard, 20 different tests are set for each EIT, which involves training a new ANN for each test.

Finally, with the aim of showing average results for each studied error under simulated and real scenarios, an averaging process over the tests and over the different EITs is performed.

**Table 4:** Error indicators for different offset error levels—Real data.

Error Level	Real Data—Offset			
	MAPE (%)	NMBE (%)	NRMSE (%)	Abs (%)
**−10 %**	12.932	0.568	16.230	0.072
**−9 %**	14.365	0.621	18.026	0.072
**−8 %**	16.153	0.681	20.268	0.071
**−7 %**	18.448	0.750	23.143	0.069
**−6 %**	21.503	0.829	26.968	0.066
**−5 %**	25.773	0.919	32.313	0.064
**+5 %**	25.576	3.271	31.930	0.146
**+6 %**	21.472	3.053	26.788	0.168
**+7 %**	18.517	2.822	23.090	0.183
**+8 %**	16.282	2.600	20.295	0.192
**+9 %**	14.527	2.394	18.103	0.198
**+10 %**	13.111	2.209	16.337	0.000

Table 5 details the results obtained when both offset and gain errors occur simultaneously. In the authors’ view, these results represent an excellent initial step toward the implementation of the system in open substation nodes for protection and security purposes.

**Table 5:** Combined error detection in real data

		Offset Error Level													
		−10%		−7.5%		−5%		0%		5%		7.5%		10%	
**Gain Error Level**	**−10%**	−8.9	−10.3	−8.9	−7.8	−8.9	−5.3	−8.8	−0.4	−8.5	4.5	−8.5	7.0	−8.5	9.5
	**−7.5%**	−6.7	−10.2	−6.7	−7.7	−6.7	−5.2	−6.6	−0.3	−6.3	4.6	−6.3	7.1	−6.3	9.5
	**−5%**	−4.5	−10.1	−4.5	−7.6	−4.5	−5.1	−4.4	−0.2	−4.1	4.7	−4.1	7.1	−4.1	9.6
	**0%**	−0.1	−9.9	−0.1	−7.4	−0.1	−4.9	0.0	0.0	0.2	4.9	0.3	7.4	0.3	9.8
	**5%**	4.2	−9.7	4.2	−7.2	4.2	−4.7	4.4	0.2	4.6	5.1	4.6	7.5	4.7	10.0
	**7.5%**	6.4	−9.6	6.4	−7.1	6.4	−4.6	6.5	0.3	6.8	5.2	6.8	7.6	6.8	10.2
	**10%**	8.5	−9.5	8.5	−6.7	8.6	−4.5	8.7	0.4	8.9	5.3	9.0	7.8	9.0	10.3

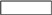
 Gain error level detected 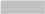 Offset error level detected

## 4. Conclusions and Future Works

Detection of measurement errors in electronic instrumental transducers (EITs) should play an important part in SS automation. Early detection of errors in the EITs can help not only reduce the risk of misoperation in the protection systems but also implement different preventive maintenance plans.

This paper has proposed a novel approach to measurement error detection based on short term load forecasting (STLF). Historical measurements acquired from EITs along with data related to the day and the month are the inputs to an ANN with the purpose of obtaining the forecast measurements for the day under consideration. Real and forecast measurements are then compared in order to independently detect offset and gain error levels.

The strength of the contribution made in this work lies in using STLF techniques to provide electricity companies with a valuable tool that serves as a decision-making aid in the area of maintenance and security. In this context, the system is designed to accurately detect offset and gain error levels introduced by EITs in SSs. This allows electricity companies to implement possible corrective and preventive actions.

Future work will concentrate on improving the forecasting model to deliver better results in terms of random errors detection and increased accuracy. With this in mind, different data, such as temperature, wind speed and economic rates could be included in the ANN training process.
